# Systemic Inflammatory Indicators as Prognosticators in Glioblastoma Patients: A Comprehensive Meta-Analysis

**DOI:** 10.3389/fneur.2020.580101

**Published:** 2020-10-07

**Authors:** Chao Yang, Hong-Bin Wen, Yu-Hang Zhao, Wen-Hong Huang, Ze-Fen Wang, Zhi-Qiang Li

**Affiliations:** ^1^Department of Neurosurgery, Zhongnan Hospital of Wuhan University, Wuhan, China; ^2^Department of Neurology, Xiangyang Central Hospital, Xiangyang, China; ^3^Department of Physiology, School of Basic Medical Science, Wuhan University, Wuhan, China

**Keywords:** neutrophils, lymphocytes, platelets, NLR, PLR, glioblastoma, overall survival

## Abstract

**Background:** Inflammation plays an important role in tumorigenesis. Previous studies have reported the prognostic value of several peripheral inflammatory markers in glioma patients, including the neutrophil-to-lymphocyte ratio (NLR). However, it still remains unclear whether inflammatory markers can independently predict the prognosis of glioblastoma (GBM) patients. The present study aims to explore the prognostic value of systemic inflammatory markers, including neutrophils, lymphocytes, platelets, the NLR, and the platelet-to-lymphocyte ratio (PLR), in patients with GBM.

**Methods:** A comprehensive systemic search and review was performed using the PubMed, EMBASE, and Cochrane Library databases to identify all the relevant literature (published before June 30, 2020) that evaluated the association between any of these inflammatory markers and survival in GBM.

**Results:** There were 2 (634 patients), 3 (723 patients), 2 (237 patients), 8 (1,225 patients), and 3 (505 patients) studies examining the correlation of survival with neutrophils, lymphocytes, platelets, the NLR, and the PLR, respectively. An elevated NLR and elevated neutrophil and platelet counts were associated with worse overall survival (OS) in GBM patients (NLR: hazard ratio [HR] = 1.63, 95% confidence interval [CI]: 1.23–2.15, *p* = 0.0007; neutrophil count: HR = 1.46, 95% CI:1.16–1.83, *p* = 0.001; platelet count: HR = 1.58, 95% CI: 1.42–1.77, *p* < 0.00001). However, there was no significant association between the PLR or the absolute lymphocyte count and OS in GBM patients.

**Conclusion:** The NLR and the absolute neutrophil and platelet counts may be valuable and convenient peripheral inflammatory markers to evaluate the prognosis of GBM patients. Further prospective studies are needed to verify its reliability.

## Introduction

Gliomas are the most common primary malignant brain tumors in adults. Glioblastoma (GBM) is the most lethal type of glioma and has a highly aggressive clinical course with a median survival time of ~14 months (1). Genome-wide molecular profiling studies have revealed many classic genetic alterations in different types of gliomas. Molecular biomarkers such as isocitrate dehydrogenase 1 (*IDH1*) and O^6^-methylguanine-DNA methyltransferase (*MGMT*) play an important role in evaluating the prognosis of glioma patients (2, 3). In the revised 2016 WHO Classification of Tumors of the central nervous system, molecular phenotypes were added to the classifications of gliomas for the first time, increasing our understanding of glioma and its prognosis (4). Although *IDH1* mutation and *MGMT* promoter methylation were already thought to be important molecular markers, disadvantages in testing techniques have impeded their widespread application. Therefore, finding additional markers for predicting the outcomes of patients with glioma that are easy to test is urgent and necessary. Neutrophil, lymphocyte, and platelet counts, as well as the neutrophil-to-lymphocyte ratio (NLR) and the platelet-to-lymphocyte ratio (PLR), can be easily obtained from routine blood examination; however, disputes on their prognostic value in GBM still exist.

Over the last decade, it has become clear that inflammation plays an important role in tumorigenesis. Long-term chronic inflammation promotes tumor initiation and progression (5), and inflammation can increase the proliferation and survival of tumor cells. The blood supply of tumors is also improved by inflammation (6). Neutrophils, lymphocytes, and platelets are classic inflammatory cells, and their levels in the peripheral blood are tightly associated with the extent of inflammation. As with other cancer patients, most glioma patients experience strong neutrophilia and lymphopenia because of overproduction of Granulocyte Colony Stimulating Factor (G-CSF) by tumor cells. G-CSF diverts bone-marrow hematopoiesis away from the lymphocyte lineage toward the granulocyte lineage (7). A review by Donskov reported that elevated neutrophils both in tumor around and in peripheral blood, and a high NLR associated with poor clinical outcomes in several human cancers, such as renal cell carcinoma, hepatocellular carcinoma, colorectal cancer, and so on (8). Especially, the significance of NLR has been largely studied in colorectal cancer. An elevated NLR was observed to independently predict poorer outcomes in advanced colorectal cancer patients with oxaliplatin-based chemotherapy (9) and with liver metastasis (10). Some reports have shown that a higher level of neutrophils predicted a worse outcome in GBM patients (11, 12). However, Lopes et al. reported that the neutrophil count does not correlate with prognosis in GBM patients (13). Most studies did not find prognostic value for the lymphocyte count in GBM, possibly because of the various subgroups of lymphocytes. However, one study reported that treatment-related lymphopenia after surgery could independently predict the prognosis of GBM patients (14). A recent review showed that thrombocytosis at the time of diagnosis is associated with shorter survival in many solid tumors, including malignant glioma (15), but other studies have reported that the platelet level cannot predict prognosis in GBM (13, 16). Previous studies have explored the prognostic value of the NLR and PLR in many cancers, including gastric cancer (17), breast cancer (18), primary liver cancer (19), advanced esophageal cancer (20), prostate cancer (21), and renal cell carcinoma (22). Compared to traditional molecular prognostic markers, such as *IDH1* mutation and *MGMT* promoter methylation status (23), these hematological inflammatory markers can conveniently evaluate the prognosis of GBM patients in order to guide clinicians' therapeutic decisions and patient management.

There have been two meta-analysises (24, 25) evaluating the prognostic role of the NLR in patients with glioma recently. However, one of them only analyzed NLR and both of them overlooked the influence of the type of variable on the final results. To our knowledge, this is the first meta-analysis to systematically assess more inflammatory markers and offer a better understanding of the prognostic value of the neutrophil, lymphocyte, and platelet counts in GBM patients.

## Materials and Methods

The Preferred Reporting Items for Systematic Reviews and Meta-Analysis (PRISMA) guidelines were used to perform this meta-analysis (26). A completed PRISMA 2009 Checklist was shown in Supplementary File 1.

### Search Strategy

We performed a systematic review to identify articles published before June 30, 2020 from the PubMed, EMBASE, and Cochrane Library databases assessing the association between any one of the five inflammatory markers and prognosis in GBM patients. We performed five searches based on different inflammatory markers. Our five searches included the Mesh Terms: (1) “NLR” and “Glioma;” (2) “PLR” and “Glioma;” (3) “Neutrophils” and “Glioma” and “Prognosis;” (4) “Lymphocytes” and “Glioma” and “Prognosis;” and (5) “Blood Platelets” and “Glioma” and “Prognosis.” The relevant free terms are listed in the Supplementary File 2. Reviews and the references of included studies were also checked to avoid omission of relevant publications.

### Eligibility Criteria

We included studies only if all the following conditions were met: (1) included patients diagnosed with GBM; (2) either preoperative or postoperative inflammatory indices (neutrophils, lymphocytes, platelets, NLR, and PLR) were evaluated using multivariate analysis; and (3) hazard ratios (HRs) and 95% confidence intervals (CIs) for overall survival (OS) were provided directly from the multivariate analysis. Studies were excluded if they met the following criteria: (1) not in humans, or (2) not published in English; (3) case reports, letters, conference abstracts and non-clinical studies; (4) duplicated publications.

### Study Selection and Data Extraction

Two reviewers (C.Y. and Y.H.Z.) independently selected relevant studies that met our inclusion criteria and extracted the relevant information, with any disagreements resolved by discussion to achieve a consensus. Publications were read for extraction of original data. The main data extracted were HRs with 95% CIs. Characteristics of the studies were also extracted, including first author, publication year, country, number of patients (female/male ratio), age, glioma grade, sampling time (before or after surgery), types of variables, and the cut-off value of inflammatory markers.

### Quality Evaluation

The Newcastle-Ottawa Scale was used to assess the quality and risk of bias of the included studies. Two authors independently conducted the assessment of the included studies based on three main aspects, including study selection (0–4 points), comparability (0–2 points), and study outcomes (0–3 points). The maximum score is nine points and high scores represent high quality and low risk of bias (Table 1).

**Table 1:** Quality evaluation of included studies using the Newcastle Ottawa Scale for cohort studies.

**Markers**	**Author**	**Year**	**Selections**				**Comparability E**	**Outcome**			**Score**
			**A**	**B**	**C**	**D**		**F**	**G**	**H**	
Neutrophil	Mason	2017	1	1	1	0	2	1	1	1	8
	Bertaut	2016	1	1	1	0	1	1	1	1	7
Lymphocyte	Lopes	2018	1	1	1	0	1	1	1	1	7
	Mason	2017	1	1	1	0	2	1	1	1	8
	Han	2015	1	1	1	0	2	1	1	1	8
Platelet	Zhou	2016	1	1	1	0	1	1	1	1	7
	Brockmann	2007	1	1	1	0	1	1	1	1	7
NLR	Mason	2017	1	1	1	0	2	1	1	1	8
	Wang PF	2017	1	1	1	0	2	1	1	1	8
	Han	2015	1	1	1	0	2	1	1	1	8
	Bambury	2013	1	1	1	0	1	1	1	1	7
	Kaya	2017	1	1	1	0	1	1	1	1	7
	Weng	2018	1	1	1	0	1	1	1	1	7
	Brenner	2019	1	1	1	0	2	1	1	1	8
	Zhang	2019	1	1	1	0	1	1	1	1	7
PLR	Wang	2017	1	1	1	0	2	1	1	1	8
	Han	2015	1	1	1	0	2	1	1	1	8
	Hao	2019	1	1	1	0	2	1	1	1	8

*A: Representativeness of the exposed cohort: Highly representative or partially representative (one point); no description (no point), B: Selection of the non-exposed cohort: Patients drawn from the same community as the exposed cohort (one point); patients drawn from a different source or no description (no point), C: Ascertainment of exposure: Information obtained from secure record or structured interview (one point); written self-report or no description (no point), D: Demonstration that outcome of interest was not present at start of study: yes (one point), no (no point), E: Comparability of cohorts based on the design or analysis: Study controls for important factor, such as age, ECOG, molecular markers, extent of resection, (two points: All factors were included, one point: Part of them were included), F: Assessment of outcome: Independent blind assessment or record linkage (one point); self-report or no description (no point), G: Follow-up long enough for outcomes to occur: Yes (one point); no (no point), H: Adequacy of follow-up of cohorts: Complete follow up-all subjects accounted for or subjects lost to follow up unlikely to introduce bias (one point); no statement (no point)*.

### Statistical Analysis

Time-to-event data (OS) was analyzed by the HR. Forest plots were drawn to obtain the pooled HR, which was considered statistically significant if the 95% CI did not overlap 1 and the *p* < 0.05. Subgroup analysis was performed based on sampling time. The heterogeneity among studies was also evaluated, with *P* < 0.10 or *I*^2^ > 50% indicating significant heterogeneity (27), and random- or fixed-effects models were adopted when the heterogeneity was or was not significant, respectively. The sources of heterogeneity were evaluated by subgroup analysis. Sensitivity analysis was performed by excluding a single study at a time to examine the stability of the results. Publication bias was evaluated by funnel plot. Statistical difference was defined as *P* < 0.05. All the statistical processes were performed using RevMan Version 5.3 (Review Manager, Copenhagen: The Nordic Cochrane Center, The Cochrane Collaboration, 2014).

## Results

### Literature Search

**Figure 1:**
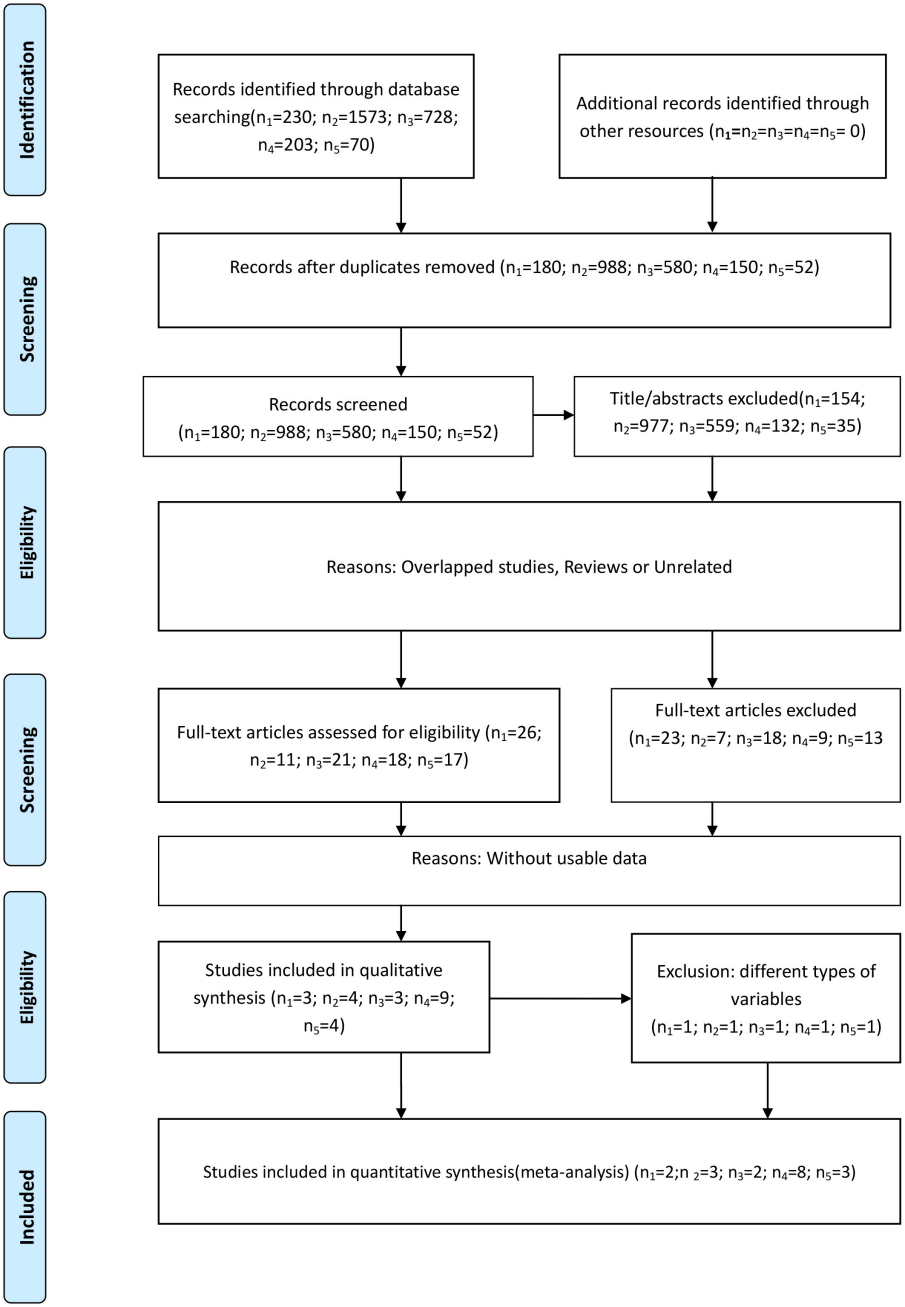
Flow diagram of the study selection process. (n_1_-n_5_ represent the numbers of incorporated literature of neutrophils, lymphocytes, platelets, NLR, and PLR, respectively).

There were 230, 1573, 728, 203, and 70 studies initially identified examining the absolute neutrophil, lymphocyte, and platelet counts and the NLR and PLR, respectively. We removed duplicated studies, and, after full-text evaluation, conference abstract, and articles lacking sufficient data were excluded. One study (13) treated these five inflammatory markers as continuous variables and was excluded in the last quantitative synthesis for these markers, except the lymphocyte count, because of differences in variable type. Likewise, one study (14) treated the lymphocyte count as a dichotomous variable, which was different from the remaining studies, and was therefore excluded in the last quantitative synthesis. Finally, there were 2 (11, 12), 3 (11, 14, 28), 2 (16, 29), 8 (11, 30–36), and 3 (30, 31, 37) included studies examining the neutrophil, lymphocyte, and platelet counts and the NLR and PLR, respectively. All the incorporated studies focused on glioblastoma patients performing the multivariate analysis. The study selection process based on the PRISMA statement (Supplementary File 1) is shown in Figure 1. The main characteristics of the incorporated studies are presented in Table 2.

**Table 2:** Characteristics of the included studies.

**Markers**	**Author**	**Year**	**Country**	**N (F/M)**	**Age**	**Grade**	**Sampling time**	**Cut-off value**	**Multivariate HR**
Neutrophils (*10^9^/L)	Mason	2017	Canada	369 (131/238)	Median 55	IV	Postoperative	8	Yes
	Bertaut	2016	France	265 (106/159)	Mean 59.8; Median 61	IV	Postoperative	6,000/mm^3^	Yes
Lymphocytes (*10^9^/L)	Lopes	2018	Portugal	140 (42/98)	Mean 62.9	IV	Preoperative	\	Yes
	Mason	2017	Canada	369 (131/238)	Median 55	IV	Postoperative	\	Yes
	Han	2015	China	214 (94/120)	Mean 52.3	IV	Preoperative	\	yes
Platelets (*10^9^/L)	Zhou	2016	China	84 (34/50)	Median 53	IV	Preoperative	151	Yes
	Brockmann	2007	Germany	153 (63/90)	\	IV	Preoperative	\	yes
NLR	Mason	2017	Canada	369 (131/238)	Median 55	IV	Postoperative	7.5	Yes
	Wang PF	2017	China	166 (70/96)	Mean 52.1	IV	Preoperative	4	Yes
	Han	2015	China	152 (57/95)	Mean 50.4	IV	Preoperative	4	Yes
	Bambury	2013	Ireland	84 (19/65)	Median 58	IV	Preoperative	4	Yes
	Kaya	2017	Turkey	90 (39/51)	Mean 55.7; Median 58.5	IV	Preoperative	5	Yes
	Brenner	2019	Israel	89 (43/46)	Median 59	IV	Preoperative	4	Yes
	Zhang	2019	China	170 (–)	\	IV	Preoperative	7.25	Yes
	Weng	2018	China	105 (52/53)	\	IV	Preoperative	4	Yes
PLR	Wang	2017	China	166 (70/96)	Mean 52.1	IV	Preoperative	175	Yes
	Han	2015	China	152 (57/95)	Mean 50.4	IV	Preoperative	135	Yes
	Hao	2019	China	187 (71/116)	Mean 55	IV	preoperative	228.6	Yes

*N (F/M): numbers of patients (female/male), Grade: the pathological grade of glioma, Sampling time: the blood was obtained preoperatively or postoperatively, HR: hazard ratio*.

### Association Between the OS and Inflammatory Markers

We performed five analyses based on the different inflammatory markers.

#### Neutrophil Count

**Figure 2:**
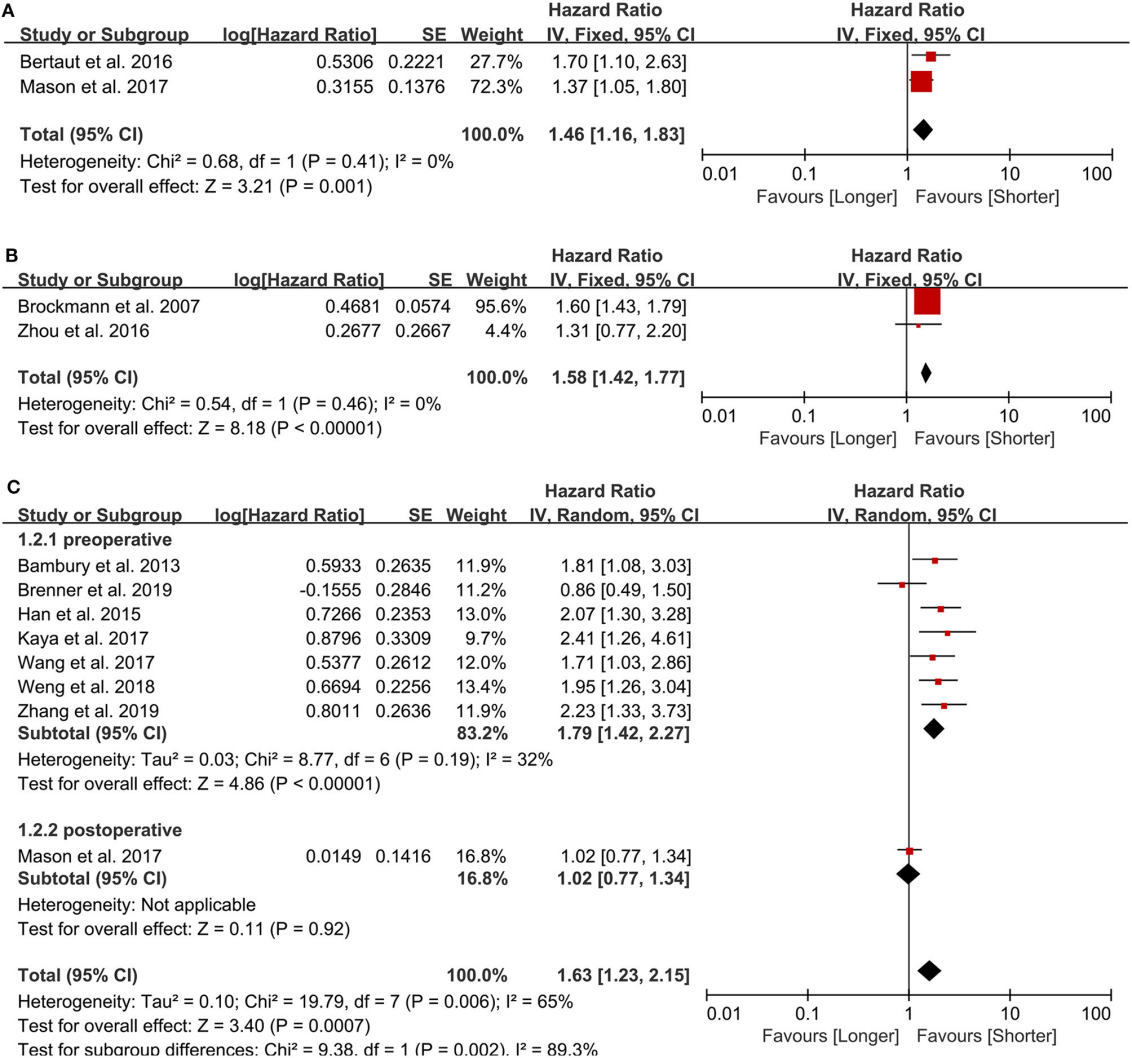
Pooled hazard ratio (HR) and 95% confidence intervals (CIs) for OS in GBM patients. **(A)** HR and 95% CIs of the count of neutrophils. An elevated neutrophil count predicted a poor prognosis in GBM patients. **(B)** HR and 95% CIs of the count of platelets. Elevated platelets also predicted a bad outcome in patients with GBM. **(C)** HR and 95% CIs of NLR. A higher NLR predicted a worse prognosis in GBM patients either in total group analysis or pre- and post-operative subgroup analysis.

Two studies examining 634 patients were included in the analysis. One study (11) from Canada was published in 2017, another from France (12) was published in 2016. The cut-off value are 8, 6 (10e9/L), respectively. Both of them analyzed the neutrophil count as category variable. The NOS scores are 8, 7 points, respectively. Both studies sampled the neutrophil count postoperatively, and an elevated neutrophil count predicted a poor prognosis in GBM patients (HR = 1.46, 95% CI: 1.16–1.83, *p* = 0.001, *I*^2^ = 0%, fixed-effect model) (Figure 2A). We did not perform sensitivity analysis because there were only two studies.

#### Platelet Count

Two studies examining 237 patients were examined for this analysis. One from China (16) was published in 2016 and another from Germany (29) was published in 2007. The cut-off value are 151, 400(10e9/L), respectively, and all the NOS scores are 7 points. Both sampled the platelet count preoperatively and evaluated it as a dichotomous variable. Elevated platelets also predicted a bad outcome in patients with GBM (HR = 1.58, 95% CI: 1.42–1.77, *p* < 0.00001, *I*^2^ = 0%, fixed-effect model) (Figure 2B).

#### NLR

We included eight retrospective studies (11, 30–36) published between 2013 and 2019 examining 1,225 patients to evaluate the association between the NLR and prognosis in GBM patients. There are 4 studies from China, 1 from Canada, 1 from Ireland, 1 from Turkey, and 1 from Israel. Five studies used 4 as the cut-off value, while the other three studies used 7.5, 7.25, and 5 as the cut-off value, respectively. The NOS of 4 studies were 8 points and the other 4 studies were 7 points. In all patients, a higher NLR predicted a worse prognosis in GBM patients (HR = 1.63, 95% CI: 1.23–2.15, *p* = 0.0007, *I*^2^ = 65%, random-effect model) (Figure 2C). Seven studies sampled preoperatively, whereas the study by Mason et al. (11) sampled postoperatively, and, in the sensitivity analysis, it was the major source of the heterogeneity. After excluding this study, the heterogeneity was 32% (*p* = 0.19), and the pooled HR remained statistically significant (HR = 1.79, 95% CI: 1.42–2.27, *p* < 0.00001).

#### Lymphocyte Count

**Figure 3:**
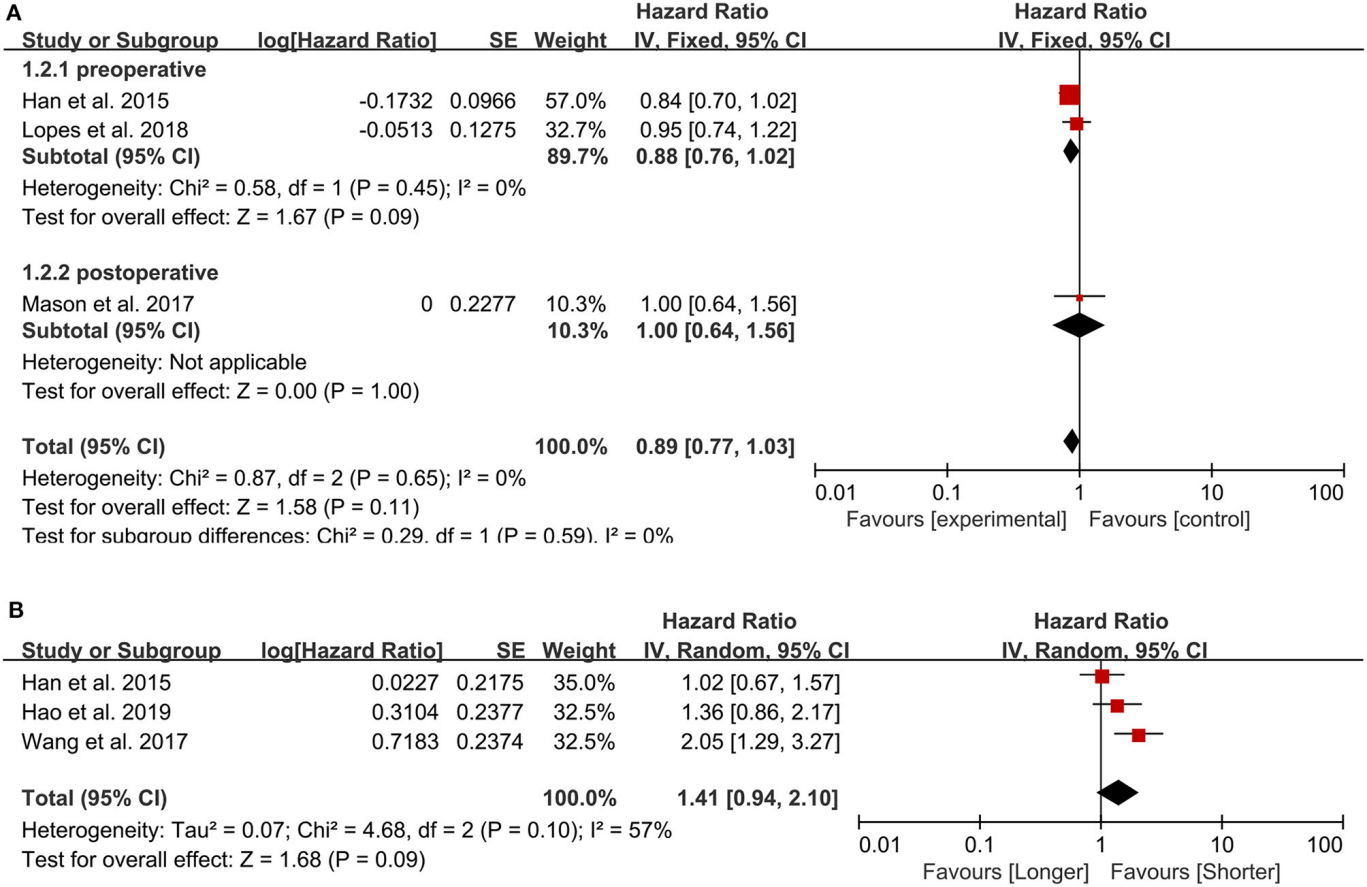
Pooled hazard ratio (HR) and 95% confidence intervals (CIs) for OS in GBM patients. **(A)** HR and 95% CIs of the count of lymphocytes. The peripheral lymphocyte count did not have prognostic value in patients with GBM both in total group analysis and subgroup analysis. **(B)** HR and 95% CIs of PLR. The PLR was not associated with the prognosis of GBM patients.

Three studies were included in this analysis, and the pooled HR for OS was 0.89 (95% CI: 0.77–1.03) (Figure 3A), indicating that the peripheral lymphocyte count did not have prognostic value in patients with GBM. Sensitivity analysis showed that the result was stable. One study from Portugal (13) was published in 2018, one from Canada (11) was published in 2017 and the last one from China (28) was published in 2015. All the incorporated studies analyzed the lymphocyte count as the continuous variable. Two studies sampled preoperatively and one sampled postoperatively. The NOS are 7, 8, 8 points, respectively. In further subgroup analysis, the pooled HR of the lymphocyte count for the two studies assessing lymphocyte count before surgery was 0.88 (95% CI: 0.76–1.02).

#### PLR

We included three studies examining 505 patients, and, in our meta-analysis, the PLR was not associated with the prognosis of GBM patients (HR = 1.41, 95% CI: 0.94–2.10, *p* = 0.09) (Figure 3B). All the three studies (30, 31, 37) published between 2015 and 2019 were from China and sampled preoperatively. The cut-off value were 175, 135, 228.6, respectively, and the NOS were all 8 points.

### Publication Bias

**Figure 4:**
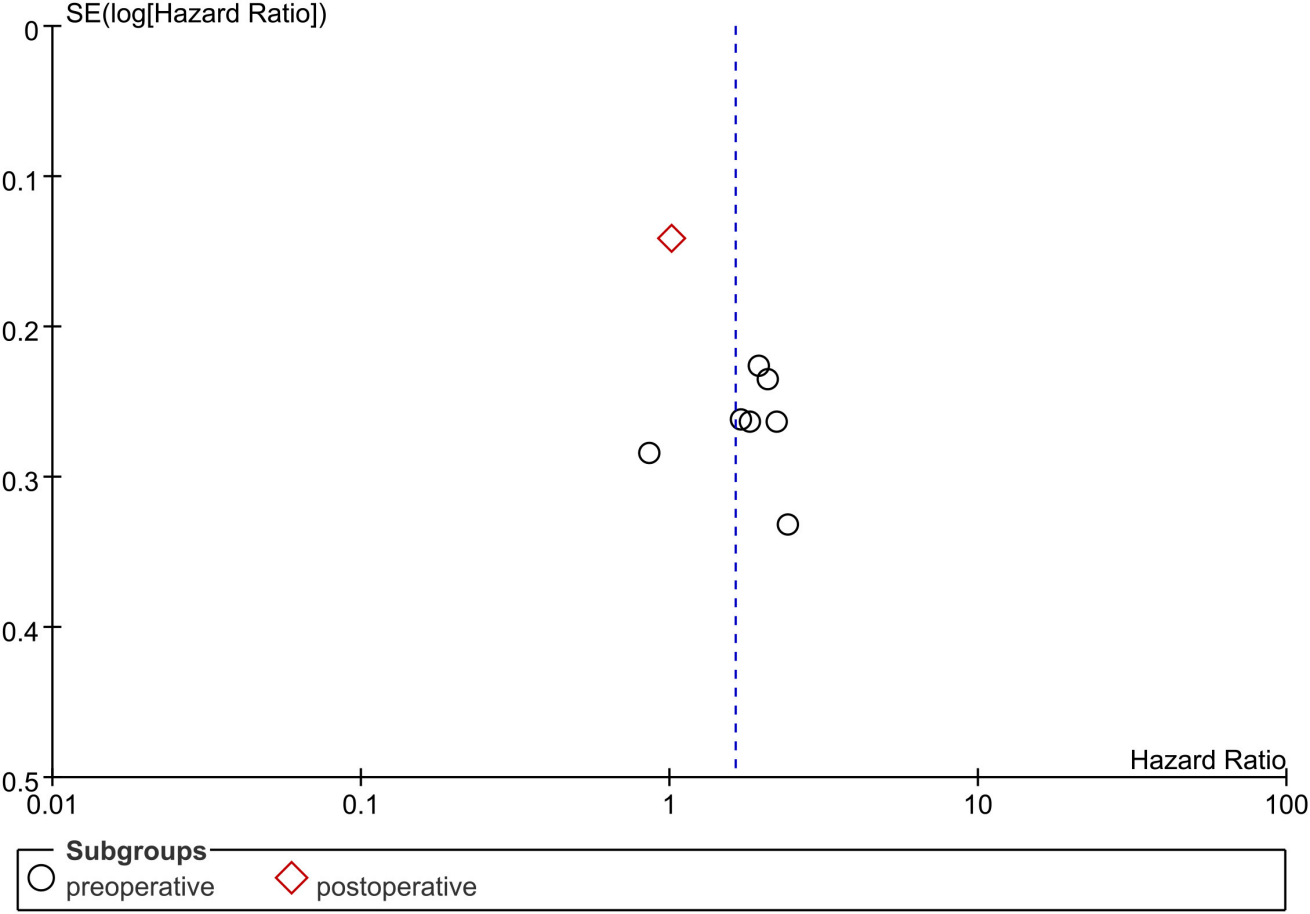
Funnel plot of publication bias of NLR in GBM patients. No significant publication bias was found in the present meta-analysis.

Due to limitations in the quantity of included studies, we only tested the publication bias in studies examining the NLR. No significant publication bias was found in the present meta-analysis (Figure 4).

## Discussion

The interplay between inflammation and cancer has been widely investigated in the past few decades. Tumor progression is greatly influenced by inflammatory cells, and inflammation may drive the secretion of some crucial pro-tumorigenic signals, including growth factors and pro-angiogenic factors (5). Inflammation may also accelerate the development of glioma (38, 39). Neutrophils, lymphocytes, and platelets and the NLR and PLR are all markers of the systemic inflammatory response. Previous studies have reported the potential prognostic value of inflammatory markers in various tumors. Importantly, peripheral inflammatory markers can be easily measured during routine blood examination after hospitalization. To our best knowledge, this is the first meta-analysis considering all the relevant peripheral inflammation indices based on peripheral blood cells to explore their use as prognostic markers in GBM patients.

In our study, the NLR and the absolute neutrophil count independently predicted the prognosis of GBM patients, showing their possible values as prognostic biomarkers in GBM. The peripheral blood neutrophil count is frequently high in cancer patients (31, 32, 40). Neutrophils may promote tumor progression by secreting important cytokines, such as VEGF (41), IL-6 (42), IL-8 (43), elastases (44), and matrix metalloproteinases (45). The extent of neutrophil infiltration is thought to significantly correlate with tumor grade in glioma, and the number of circulating or infiltrating neutrophils around the tumor may be influenced by factors deriving from glioma (46). Tumor cells may increase the neutrophil count both in the peripheral blood and around the tumor by secreting chemotactic factors, such as G-CSF, VEGF, IL-1β, and IL-6 (40, 47, 48). A recent study also reported that the blood neutrophil count may predict the efficacy of bevacizumab in GBM (12). An increased neutrophil count is also thought to inhibit lymphocyte survival and suppress the normal cytotoxic function by regulating lymphocyte function via releasing reactive oxygen species and arginase (40). The two studies included in this meta-analysis that analyzed the neutrophil count analyzed it as a categorical variable and used postoperative blood samples. As a result, most patients have likely already been exposed to corticosteroids, which affects both the neutrophil and lymphocyte counts, and the stress from surgery itself may also have an impact on systemic inflammation (31). However, the lymphocyte count was not associated with the prognosis of GBM patients in our study. Additionally, the two studies did not state at which time point after surgery they collected their blood samples, and the cut-off values were different. Interestingly, the decrease rate in the neutrophil count has also been reported to correlate with survival in GBM patients. A decrease rate of 40% or higher markedly predicts good prognosis of *IDH*-wild type GBM patients in the concomitant TMZ phase (49). Therefore, caution should be applied to this finding, and more studies are needed to verify it.

The NLR, by comprising the neutrophil and lymphocyte counts, is regarded as an index that reflects the balance of the inflammation and immune responses. A meta-analysis performed by Templeton et al. (50) indicated that a higher NLR predicted a poorer OS in many solid tumors. In this study, five studies used an NLR of 4 as the cut-off value, and the pooled result was consistent with previous findings. One study by Kaya et al. used 5 as the cut-off value and found that the NLR was correlated with survival (33), whereas another study by Mason et al. used 7.5 as the cut-off value and found that the NLR was not correlated with survival (11), although they used postoperative samples. Notably, a recent study by Brenner et al. (35) come to the result that the pretreatment NLR did not have the prognostic value in glioblastoma patients treated with combined modality surgery, radiation, and temozolomide, indicating the prognostic significance of the NLR in some designate cohort remains unclear. The NLR was also found to positively correlate with the expression of Ki-67 (34) and the glioma grade (51–53). A more exact conclusion from our study may be that a preoperative NLR >4 may predict worse outcome in GBM patients, and the postoperative neutrophil count may be a marker for the prognosis of GBM patients. Nevertheless, we recommend that future studies use preoperative blood samples in order to avoid the potential factors mentioned above. The exact mechanisms by which the NLR influences the prognosis of GBM patients remains unknown.

Another marker of systemic inflammation is the PLR (54), which has also been studied as a prognostic indicator in many cancers, including non-small cell lung cancer (55) and renal cell carcinoma (22). The underlying mechanism of the PLR in GBM is still confusing. An elevated platelet level may boost tumor growth, angiogenesis, and dissemination (56) by secreting crucial factors, such as VEGF (57–59). To our knowledge, this study is the first to evaluate the important roles of the platelet count in glioma cohorts. According to our results, an elevated preoperative platelet count predicts a poorer prognosis in GBM patients. However, the two studies used different cut-off values, which may influence the clinical utility of this finding. More studies are needed to further explore the prognostic value of the preoperative platelet count. However, in this study, the PLR showed no prognostic value in GBM patients, and the heterogeneity was significant (*I*^2^ = 57%). Therefore, caution should be taken in applying this result.

In this study, assessing the neutrophils, lymphocytes, platelets, NLR, and PLR as either categorical or continuous variables may have had an effect on the results. Lopes et al. (13) evaluated these factors as continuous variables and came to the conclusion that none of these markers had prognostic value in GBM patients. Because the other included studies evaluated these markers as categorical variables, this study was excluded from the quantitative synthesis in the analysis of neutrophils, platelets, the NLR, and the PLR. Unlike the other three studies assessing the lymphocyte count, Mendez et al. (14) treated the lymphocyte count as a dichotomous variable, and therefore it was excluded from the quantitative synthesis. We recommend that future studies take notice of the influence of variable types involving different statistical methods on results.

There are also some limitations in our meta-analysis. First, nearly all of the incorporated studies were retrospective, which inevitably causes some bias due to the nature of retrospective analysis. Second, there were only a small number of studies of some markers; therefore, the results should be applied with caution. Moreover, the characteristics among studies varied considerably, including the patients with different race or from different regions, which may be an important source of heterogeneity. More optimally designed studies are needed to validate our results. Although there has been a meta-analysis evaluating the prognostic significance of the NLR in glioma patients, our study focused on the cohort of glioblastoma and included more systemic inflammatory indicators and more independent studies on the NLR in glioblastoma patients, and is therefore valuable, despite these limitations.

## Conclusions

In conclusion, the NLR and the neutrophil and platelet counts may be valuable and convenient peripheral inflammatory markers to evaluate the prognosis of GBM patients. However, more prospective and large-scale studies are needed to verify our results. The complex relationship between the GBM microenvironment and systemic inflammatory responses should be profoundly investigated in order to clarify the mechanisms of these inflammatory markers in GBM.

## Data Availability Statement

The raw data supporting the conclusions of this article will be made available by the authors, without undue reservation.

## Ethics Statement

The studies involving human participants were reviewed and approved by Ethics Committee of Zhongnan Hospital of Wuhan University. The patients/participants provided their written informed consent to participate in this study.

## Author Contributions

CY: took responsibility for the integrity of the data and the accuracy of the data analysis. CY and H-BW: drafted the manuscript. CY and Y-HZ: statistical analysis. CY, H-BW, Y-HZ, and W-HH: critical revision of the manuscript for important intellectual content. Z-FW and Z-QL: supervision. All authors: concept, design, analysis and interpretation of data.

## Conflict of Interest

The authors declare that the research was conducted in the absence of any commercial or financial relationships that could be construed as a potential conflict of interest.
