# Supplementary material for: Systemic Inflammatory Indicators as Prognosticators in Glioblastoma Patients: A Comprehensive Meta-Analysis
Source: Front Neurol. 2020 Oct 7;11:580101. doi: 10.3389/fneur.2020.580101 (PMC7575748; doi:10.3389/fneur.2020.580101)
Supplement: Supplementary file 2 [file Data_Sheet_2.PDF]

Free terms:

(1) “Gliomas”, “Glial Cell Tumors”, “Glial Cell Tumor”, “Tumor, Glial Cell”, “Tumors, Glial Cell”, “Mixed Glioma”, “Glioma, Mixed”, “Gliomas, Mixed”, “Mixed Gliomas”, “Malignant Glioma”, “Glioma, Malignant”, “Gliomas, Malignant”, “Malignant Gliomas”; (2) “neutrophil to lymphocyte ratio”, “neutrophil-lymphocyte ratio”; (3) “platelet to lymphocyte ratio”, “platelet-lymphocyte ratio”; (4) “Neutrophil”, “Leukocytes, Polymorphonuclear”, “Leukocyte, Polymorphonuclear”, “Polymorphonuclear Leukocyte”, “Polymorphonuclear Leukocytes”, “LE Cells”, “Cell, LE”, “Cells, LE”, “LE Cell”, “Neutrophil Band Cells”, “Band Cell, Neutrophil”, “Band Cells, Neutrophil”, “Neutrophil Band Cell”; (5) “Lymphocyte”, “Lymphoid Cells”, “Cell, Lymphoid”, “Cells, Lymphoid”, “Lymphoid Cell”; (6) “Blood Platelet”, “Platelet, Blood”, “Platelets, Blood”, “Thrombocytes”, “Thrombocyte”, “Platelets”, “Platelet”; (7) “Prognoses”, “Prognostic Factors”, “Factor, Prognostic”, “Factors, Prognostic”, Prognostic Factor”.
